# Vitamin B_12_ intake during pregnancy linked to child speech development and intelligence quotient

**DOI:** 10.1093/pubmed/fdae307

**Published:** 2024-12-14

**Authors:** Eliska Hrezova, Gabriela Ksinan Jiskrova, Tomas Prusa, Lenka Andryskova, Hynek Pikhart

**Affiliations:** RECETOX, Faculty of Science, Masaryk University, Brno 625 00, Czech Republic; RECETOX, Faculty of Science, Masaryk University, Brno 625 00, Czech Republic; Department of Public Health, Faculty of Medicine, Masaryk University, Brno 625 00, Czech Republic; RECETOX, Faculty of Science, Masaryk University, Brno 625 00, Czech Republic; RECETOX, Faculty of Science, Masaryk University, Brno 625 00, Czech Republic; Department of Epidemiology and Public Health, University College London, Institute of Epidemiology and Health Care, London WC1E 7HB, UK

**Keywords:** children, epidemiology, food and nutrition

## Abstract

**Background:**

Nutrient deficiencies during pregnancy may affect offspring development. We aim to examine the association between prenatal vitamin B_12_ intake and children’s cognitive development.

**Methods:**

A total of 5151 mother–child pairs from the Czech part of ELSPAC study were included in the analysis. Dietary information was obtained during pregnancy using food frequency questionnaire. Parents reported on their child’s speech and language development at 18 months, 3, 5 and 7 years. Intelligence quotient (IQ) was measured at 8 years in subcohort of 854 children.

**Results:**

Children of mothers with higher vitamin B_12_ intake demonstrated higher scores in language (*B* = 0.20, 95% CI 0.06, 0.34) and talking and understanding (*B* = 2.39, 95% CI 0.97, 3.80) in a fully adjusted model at 18 months. Additionally, they were more likely to get maximum points in the intelligibility test at age 3 (OR = 1.05, 95% CI 1.01, 1.09) in unadjusted model, however, not in fully adjusted model. We found a positive effect of higher vitamin B_12_ intake on verbal IQ (*B* = 1.08, 95% CI 0.09, 2.08).

**Conclusions:**

We identified consistent associations between prenatal vitamin B_12_ intake and children’s cognitive development. The results suggest that inadequate vitamin B_12_ during pregnancy may negatively affect children’s cognitive development, particularly in speech and language.

## Introduction

Maternal diet during pregnancy is a key determinant of the offspring’s prenatal development. Deficiencies in nutrient intake may have an effect on children’s health outcomes and their neurocognitive development.^1–4^ While the effects of several nutrients, e.g. folic acid and iron or iodine have been well described before,^5^^,^^6^ the evidence about prenatal maternal vitamin B_12_ intake and children’s neurocognitive development is inconsistent.^5^

Vitamin B_12_ is necessary for intrauterine foetal development, particularly for the nervous system during childhood. It contributes to axon myelination, essential for impulse conduction from cell to cell, and protects neurons from degeneration.^7^ Vitamin B_12_ also supports brain growth, neurogenesis and synaptic connectivity, especially in the auditory and visual cortices.^8^ Disruptions in myelination can significantly impact central nervous system function later in childhood, resulting in slower conduction in the auditory and visual systems, which can interfere with learning and social interactions.^9–11^ Additionally, vitamin B_12_ is active in the metabolism of both fatty acids and amino acids^12^ and is a required cofactor in one-carbon metabolism; its deficiency leads to elevated levels of homocysteine.^13^ Elevated homocysteine levels during pregnancy may cause adverse outcomes in offspring, such as lower scores in expressive language and gross motor domains.^14^ Therefore, vitamin B_12_ is essential for the normal function of the nervous system and may potentially impact memory, language and visual and auditory processing in the child.

Several studies from diverse populations described associations between mother’s vitamin B_12_ intake during pregnancy and cognitive and language outcomes of their children with mixed results. For example, a recent study from northern Spain found that medium vitamin B_12_ levels in the first trimester were associated with better infant motor, language and cognitive performance 40 days after birth^15^ and higher working memory scores in 4 years old children.^16^ Additionally, data from the Avon Longitudinal Study of Parents and Children (ALSPAC) study showed that children of mothers with low prenatal vitamin B_12_ intake had reduced ability in speech, language and mathematics in childhood.^17^ According to systematic review, observational studies demonstrated an association between low maternal vitamin B_12_ status and worse longer-term cognitive functioning.^13^ Not all studies, however, show consistent results. In a study of US mothers and children, maternal vitamin B_12_ intake from food and supplements was negatively associated with offspring’s receptive language at age 3 years^18^ with no effect observed by age 7.^19^ Similarly, Wu *et al*.^20^ found no association with children’s language, cognitive and motor skills at the age of 1.5 in a Canadian study.

Given the inconsistent evidence in the literature, the objective of our study was to analyze the association between maternal vitamin B_12_ intake during pregnancy and the cognitive outcomes of children, especially language outcomes in a longitudinal study of children from Central Europe where such analysis has not been conducted to date.

## Methods

### Study sample

European Longitudinal Study of Pregnancy and Childhood (ELSPAC) is a population-based prospective longitudinal birth cohort study. The study was initiated by the World Health Organization for Europe in 1985 and coordinated by Bristol University (ALSPAC) to collect data across Europe. In the Czech part of ELSPAC study (ELSPAC-CZ), pregnant women with residence in the Brno and Znojmo regions and with expected delivery between 1 March 1991 and 30 June 1992 were enrolled. Information on 5151 mother–child pairs was available.^21^

Parents filled out self-reported questionnaires about health, lifestyle, dietary habits, demographic, psychosocial factors and environmental exposures about themselves and their child before birth, and 6 months, 18 months, and 3, 5, 7, 11, 15 and 19 years after birth. All participants in the study were invited to participate in psychological assessments, including IQ testing at age 8. Those who were willing and able to attend went through the psychological examination done by trained psychologists. We used questionnaire data from the prenatal period, the 18 months after birth, and at ages 3, 5 and 7 years. Further, we used data from psychological examinations done on the subsample of 854 children at 8 years.

### Dietary intake

**Fig. 1:**
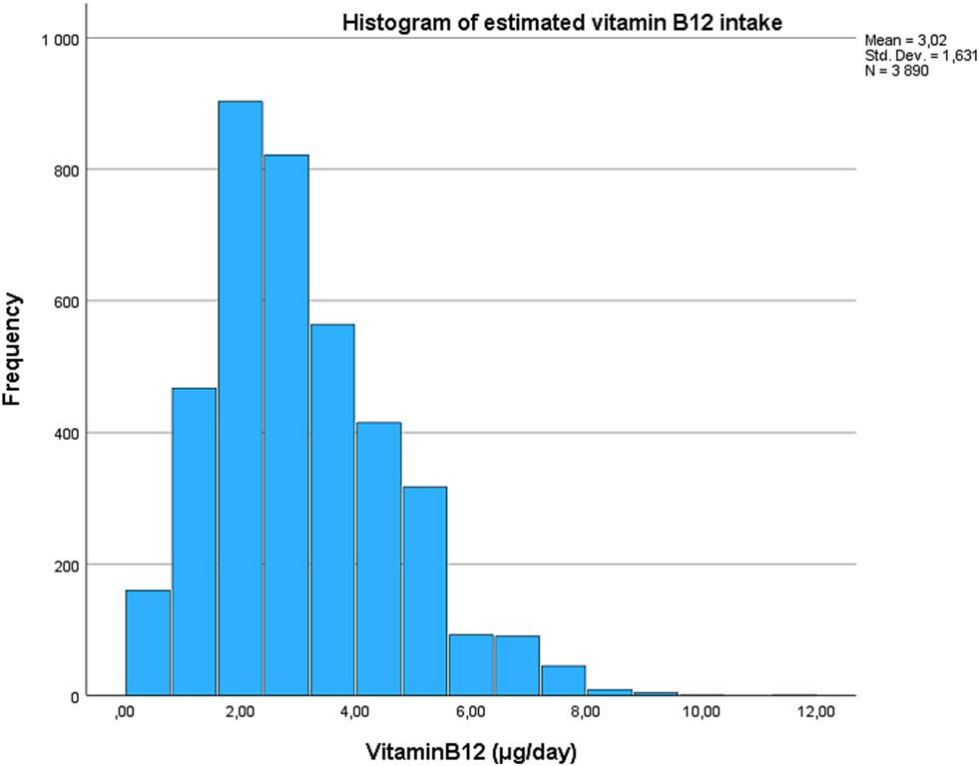
Histogram of estimated vitamin B_12_ intake for initial sample.

**Fig. 2:**
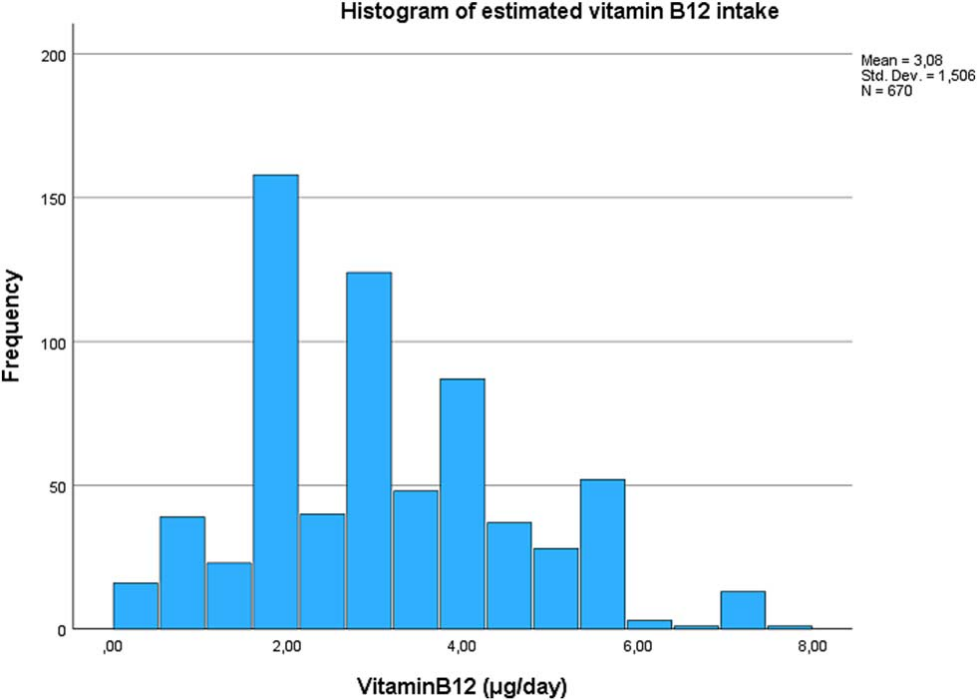
Histogram of estimated vitamin B_12_ intake for subsample.

To estimate maternal prenatal vitamin B_12_ intake in the ELSPAC-CZ study, we used the food frequency questionnaire (FFQ) data. Self-reported FFQ with 145 items in 36 separate food groups was administered at 32 weeks of gestation. On a five-point scale from *never or rarely* to *more than once a day*, women reported how frequently they consumed particular foods or drinks during their pregnancy. We used 17 food groups to estimate vitamin B_12_ intake (Supplementary Table S1) and excluded those where vitamin B_12_ does not naturally occur based on the methodology described before.^22^ Briefly, individual food items within each food group were combined with vitamin B_12_ content data from national databases and reported as μg per 100 g or 100 ml.^23–25^ A weighted average of portion size was estimated for each food group, and total daily vitamin B_12_ intake was calculated by multiplying the frequency of consumption by the quantity of vitamin B_12_ per 100 g/ml and portion size (Figs 1 and 2).

### Outcomes

#### Speech and language

At the 18th month of children’s age, several domains of speech and language development were measured by adapted questions from Denver scale^26^ (language—word combination, use of plurals and negative sentences), and MacArthur Infant Communication questionnaire^27^ (talking and understanding). Intelligibility (referring to how well are children understood) was measured at 3, 5 and 7 years of children’s age. The scales were back translated from the English original to Czech language and their cultural appropriateness was evaluated via a pilot study prior to the data collection.^28^

#### Language

A group of 12 questions related to elementary expressions (e.g. She/he says mom and dad, colors, her/his name), word combinations, plurals and negatives were answered on the following scale: 0—no, does not yet; 1- yes, once or twice; 2-yes, very good. The total language score was calculated as a sum of all items.

### Talking and understanding

A list of 134 words divided into 10 categories (sounds of animals, animals, vehicles, food and drinks, clothes, body parts, rooms and house equipment, outdoor, activity and communication, characteristics and feelings) was provided in the questionnaire. Vocabulary knowledge of a child was reported on the following scale: 0—no does not say yet; 1—understands but does not say; 2—says and understands. Early communication (e.g. pointing with a finger, waving to say goodbye) was measured with a group of 10 questions with possible answers on a scale: 0—no, does not yet; 1—sometimes; 2—often. To measure understanding, parents were asked a total of 13 questions: *Which of these following questions does your child understand* (e.g. Are you hungry?, Come here.) and possible answers were recorded on a scale: 0—no; 1—yes. The talking and understanding score was calculated as a sum of all items.

### Intelligibility

Mothers answered three single questions *Do* (1) *you,* (2) *family and* (3) *visitors understand what the child says*? using a scale a scale: 0—rarely; 1—mostly; 2—sometimes at 3 years of age and: 0—never, 1—sometimes, 2—often and 3—always at 5 and 7 years of age, respectively. Intelligibility scores for each age were calculated as a sum. Due to the highly left skewed distribution of the data, we classified the intelligibility scores into binary variables with those who got maximum points and those who did not.

### IQ

The individual psychological examination of children was carried out on the subset of the sample. IQ was measured by Wechsler Intelligence Scale for Children III (WISC-III) at 8 years of age.^29^ Performance and verbal IQ scores were calculated using 10 of the original 12 subtests of WISC-III.

### Covariates

Birth and pregnancy covariates considered in the analysis included sex of the child (male; female), birthweight (<2500 g; ≥2500 g), head circumference, mother’s age, mother’s body mass index (BMI) prior to pregnancy, alcohol consumption during the first 3 months of pregnancy (yes; no), smoking status during the pregnancy (non-smoker; ex-smoker; smoker), total energy intake and use of dietary supplements during pregnancy. Further socio-demographic and lifestyle factors of mothers and fathers collected during pregnancy were included: maternal education (primary; vocational; secondary; university), paternal education (primary; vocational; secondary; university), number of other children younger than 15 years of age in the same household and number of adults (including mother) over 18 years of age in the same household. Additionally, factors reported at the child’s 18-month follow-up included breastfeeding (yes—ever or still breastfed; no—never breastfed), and if the mother worked at 18th month of children’s age (yes; no).

**Table 1:** Maternal, paternal and children’s descriptive characteristics of the initial and imputed sample

Variable	*N* = 5151 Original initial sample		*N* = 5151 Imputed sample	
	*N*	Mean (SD) or valid %	*N*	Mean (SD) or %
Vitamin B_12_ intake (μg/d)	3890	3.0 (1.6)	5151	3.1 (1.6)
Maternal age (years)	5144	24.8 (4.8)	5151	24.7 (4.8)
BMI prior to pregnancy (kg/m^2^)	3760	22.1 (3.3)	5151	22.1 (3.3)
Head circumference (cm)	4912	34.6 (1.4)	5151	34.6 (1.4)
Mean energy intake (MJ)	3890	5.9 (2.2)	5151	5.9 (2.2)
Sex of the child				
Female	2493	48.4	2495	48.4
Male	2655	51.6	2656	51.6
Missing	3			
Birthweight				
<2500 g	266	5.3	274	5.3
≥2500 g	4765	94.7	4877	94.7
Missing	120			
Education—mother				
Primary	282	7.1	386	7.5
Vocational	1359	34.4	1808	35.1
Secondary	1588	40.2	2047	39.7
University	722	18.3	910	17.7
Missing	1200			
Education—father				
Primary	210	5.3	295	5.7
Vocational	1801	45.6	2377	46.1
Secondary	917	23.2	1190	23.1
University	1018	25.9	1289	25.0
Missing	1205			
Alcohol consumption during the first 3 months of pregnancy				
No	2673	69.2	3567	69.2
Yes	1192	30.8	1584	30.8
Missing	1286			
Smoking status during the pregnancy				
Non-smoker	2289	58.6	3003	58.3
Ex-smoker	1301	33.3	1719	33.4
Smoker	317	8.1	429	8.3
Missing	1244			
Dietary supplements during the pregnancy				
Yes	1986	51.4	2617	50.8
No	1876	48.6	2534	49.2
Missing	1289			
Adults (including mother) in the same household				
0–1	177	4.5	251	4.9
2	2474	63.0	2746	53.3
3	495	12.6	940	18.2
≥4	781	19.9	1214	23.6
Missing	1224			
Other children in the same household				
0	1734	45.3	2498	48.5
1	1642	42.9	2109	40.9
2	363	9.5	448	8.7
≥3	91	2.3	96	1.9
Missing	1321			
Working status of mother^a^				
No	3202	93.5	4718	91.6
Yes	222	6.5	433	8.4
Missing	1727			
Breast feeding status^a^				
Yes	3146	91.9	4828	93.7
No	278	8.1	323	6.3
Missing	1727			
Test				
18-month language	3451	12.8 (5.2)	5151	12.5 (5.0)
18-month talking and understanding	3410	162.3 (54.4)	5151	158.7 (53.1)
3-year intelligibility	3498	5.6 (0.8)	5151	5.4 (0.8)
5-year intelligibility	3404	7.7 (2.5)	5151	7.3 (2.4)
7-year intelligibility	3092	7.9 (2.6)	5151	7.3 (2.5)

a Reported at the child’s 18-month follow-up*.*

### Analytical sample and multiple imputation of missing data

The initial sample for outcomes from 18 months to 7 years of child’s age consisted of 5151 women and children who entered the study and had completed the prenatal questionnaire, and at least one postnatal questionnaire. Within this cohort, missing data ranged from 0.1% to 40.0% across all study variables (Table 1). Within a subcohort of 854 children for whom IQ measurements at the age of 8 were available, missing data ranged from 0.5 to 23.7% (Table 2). To handle missing data, Markov chain Monte Carlo method was applied. The presented results are pooled estimates from 50 imputed data sets.

**Table 2:** Maternal, paternal and children’s descriptive characteristics of the IQ subsample and imputed sample

Variable	*N* = 854 Original subsample		*N* = 854 Imputed subsample	
	*N*	Mean (SD) or valid %	*N*	Mean (SD) or %
Vitamin B_12_ intake (μg/d)	670	3.1 (1.5)	854	3.1 (1.5)
Maternal age (years)	850	26.0 (4.9)	854	26.1 (4.9)
BMI prior to pregnancy (kg/m_2_)	654	22.0 (3.2)	854	22.2 (3.3)
Head circumference (cm)	815	34.4 (1.3)	854	34.4 (1.3)
Mean energy intake (MJ)	670	5.9 (1.9)	854	5.9 (1.9)
Sex of the child				
Female	417	48.8	417	48.8
Male	437	51.2	437	51.2
Birthweight				
<2500 g	44	5.3	45	5.3
≥2500 g	790	94.7	809	94.7
Missing	20			
Education—mother				
Primary	19	2.9	51	5.9
Vocational	163	24.5	202	23.7
Secondary	320	48.1	386	45.2
University	163	24.5	215	25.2
Missing	189			
Education—father				
Primary	17	2.6	68	8.0
Vocational	262	39.3	315	36.9
Secondary	157	23.6	200	23.4
University	230	34.5	271	31.7
Missing	188			
Alcohol consumption during the first 3 months of pregnancy				
No	447	67.1	511	59.8
Yes	219	32.9	343	40.2
Missing	188			
Smoking status during the pregnancy				
Non-smoker	422	63.2	504	59.0
Ex-smoker	201	30.1	266	31.2
Smoker	45	6.7	84	9.8
Missing	186			
Dietary supplements during the pregnancy				
Yes	420	62.9	532	62.3
No	248	37.1	322	37.7
Missing	186			
Adults (including mother) in the same household				
0–1	30	4.4	43	5.0
2	469	70.1	513	60.1
3	61	9.2	128	15.0
≥4	109	16.3	170	19.9
Missing	185			
Other children in the same household				
0	294	45.1	401	47.0
1	285	43.7	361	42.2
2	54	8.3	71	8.3
≥3	19	3.0	21	2.5
Missing	202			
Working status of mother^a^				
No	732	92.9	790	92.5
Yes	57	7.1	64	7.5
Missing	54			
Breastfeeding status^a^				
Yes	744	92.7	789	92.4
No	59	7.3	65	7.6
Missing	51			
Test				
8-year IQ verbal	854		104 (16.8)	
8-year IQ perform	854		106 (16.9)	
8-year IQ total	854		106 (16.2)	

a Reported at the child’s 18-month follow-up*.*

### Statistical analysis

To examine the association between prenatal vitamin B_12_ intake and children’s speech and language development at 18 months, linear regression models were applied on study outcomes. Logistic regression was used to address intelligibility outcomes, and linear regression was performed to test the association between vitamin B_12_ intake and children’s IQ on sample subset with available IQ data. Vitamin B_12_ was treated as a continuous variable as well as a categorical variable split into quartiles. The fourth quartile (the highest vitamin B_12_ intake) was a reference category. We tested the associations between vitamin B_12_ intake and selected outcomes in non-adjusted Model 0 and two multivariable models. Model 1 adjusted for mother’s age, mother’s BMI prior to pregnancy, sex of the child, birthweight and head circumference, and Model 2 further adjusted for smoking and alcohol intake during pregnancy, total energy intake and dietary supplement use during pregnancy, maternal and paternal education, number of children younger than 15 years of age in the same household, number of adults over 18 years of age in the same household, breastfeeding reported at 18th month of children’s age and if the mother worked at 18th month of children’s age. Statistical analysis was conducted using IBM SPSS Statistics 29 with a selected significance level of 0.05.

## Results

### Descriptives

Characteristics of the original study sample of 5151 mother–child pairs and a subsample of 854 children at 8 years old, including descriptive statistics of speech and language outcomes, as well as IQ are presented in Tables 1 and 2.

### Speech and language

Both scores were positively associated with vitamin B_12_ intake in all models (Table 3). Children of mothers with higher vitamin B_12_ intake scored higher in language (*B* = 0.20, 95% CI 0.06, 0.34) and talking and understanding (*B* = 2.39, 95% CI 0.97, 3.80) in fully adjusted models (Model 2). Complete results showing fully adjusted models with all covariates are presented in Supplementary Table S2.

**Table 3:** The effect of vitamin B_12_ intake on speech and language tests outcomes in children

Test	Model 0		Model 1		Model 2	
	*B* [95% CI]	*P* value	*B* [95% CI]	*P* value	*B* [95% CI]	*P* value
18-month language	0.11 [0.01; 0.22]	0.035	0.13 [0.03; 0.24]	0.013	0.20 [0.06; 0.34]	0.006
18-month talking and understanding	1.41 [0.27; 2.56]	0.016	1.64 [0.51; 2.77]	0.005	2.39 [0.97; 3.80]	<0.001

Model 0 unadjusted. Model 1 adjusted for mother’s age, mother’s BMI prior to pregnancy, sex of the child, birthweight and head circumference. Model 2 further adjusted for alcohol consumption during the first 3 months of pregnancy, smoking status during the pregnancy, total energy intake and dietary supplement use during pregnancy, maternal and paternal education, number of children younger than 15 years of age in the same household, number of adults over 18 years of age in the same household, breast feeding reported at 18th month of children’s age and if the mother worked at 18th month of children’s age.

A positive association was observed with intelligibility at 3 years of age in non-adjusted Model 0 (OR = 1.05, 95% CI 1.01, 1.09), however, not in fully adjusted Model 2 (OR = 1.03, 95% CI 0.99, 1.07). There were no associations at later ages in all models (Table 4).

**Table 4:** The effect of vitamin B_12_ intake on intelligibility tests outcomes in children

Test	Model 0		Model 1		Model 2	
	OR [95% CI]	*P* value	OR [95% CI]	*P* value	OR [95% CI]	*P* value
3-year intelligibility	1.05 [1.01; 1.09]	0.021	1.04 [1.00; 1.08]	0.079	1.03 [0.99; 1.07]	0.199
5-year intelligibility	1.02 [0.98; 1.06]	0.369	1.01 [0.97; 1.05]	0.651	1.01 [0.97; 1.05]	0.800
7-year intelligibility	1.02 [0.98; 1.06]	0.330	1.01 [0.97; 1.05]	0.581	1.01 [0.97; 1.05]	0.731

Model 0 unadjusted. Model 1 adjusted for mother’s age, mother’s BMI prior to pregnancy, sex of the child, birthweight and head circumference. Model 2 further adjusted for alcohol consumption during the first 3 months of pregnancy, smoking status during the pregnancy, total energy intake and dietary supplement use during pregnancy, maternal and paternal education, number of children younger than 15 years of age in the same household, number of adults over 18 years of age in the same household, breast feeding reported at 18th month of children’s age and if the mother worked at 18th month of children’s age.

### IQ

The total IQ as well as results in IQ subtests at 8 years of age was positively associated with maternal vitamin B_12_ intake in non-adjusted models and Model 1 (Table 5). The results of the fully adjusted model (Model 2) showed vitamin B12 intake significantly associated only with verbal (*B* = 1.08, 95% CI 0.09, 2.08) IQ score. The association with other covariates in a fully adjusted model is shown in Supplementary Table S3.

**Table 5:** The effect of vitamin B_12_ intake on intelligence tests outcomes in children

Test	Model 0		Model 1		Model 2	
	*B* [95% CI]	*P* value	*B* [95% CI]	*P* value	*B* [95% CI]	*P* value
8-year IQ verbal	1.37 [0.53; 2.21]	0.001	1.40 [0.58; 2.23]	<0.001	1.08 [0.09; 2.08]	0.033
8-year IQ perform	1.09 [0.23; 1.94]	0.013	1.15 [0.33; 1.97]	0.006	0.46 [−0.52; 1.44]	0.360
8-year IQ total	1.20 [0.38; 2.02]	0.004	1.25 [0.47; 2.04]	0.002	0.79 [−0.15; 1.73]	0.100

Model 0 unadjusted. Model 1 adjusted for mother’s age, mother’s BMI prior to pregnancy, sex of the child, birthweight and head circumference. Model 2 further adjusted for alcohol consumption during the first 3 months of pregnancy, smoking status during the pregnancy, total energy intake and dietary supplement use during pregnancy, maternal and paternal education, number of children younger than 15 years of age in the same household, number of adults over 18 years of age in the same household, breast feeding reported at 18th month of children’s age and if the mother worked at 18th month of children’s age.

To verify the robustness of our results and considering the right-skewed distribution of vitamin B_12_ intake, we also conducted an analysis using quartiles of B12 intake. The results (Supplementary Tables S4, S5 and S6) were consistent with those obtained using B12 as a continuous predictor, confirming that lower maternal B12 intake is associated with poorer outcomes in children’s speech and IQ scores. This trend was statistically significant for language, talking and understanding outcomes at 18 months and for verbal and total IQ in fully adjusted models at age 8 years. Notably, the lowest quartile was significantly associated with poorer outcomes compared to the highest quartile (used as the reference).

## Discussion

### Main finding of this study

In this study, we analysed the effect of maternal prenatal vitamin B_12_ intake on the language and cognitive development of offspring. We examined various outcomes, including speech and language development reported by parents and IQ measured during individual psychological examinations. Firstly, we found that higher maternal vitamin B_12_ intake during pregnancy was positively linked to higher speech and language scores in children at 18 months of age. However, while no further significant associations were identified in the domain of intelligibility tests, we still noted significant associations with IQ, particularly the verbal subtest, at 8 years of age for children whose mothers had higher vitamin B_12_ intake. This trend was observed both for vitamin B_12_ treated as a continuous and categorical variable.

### What is already known on this topic

Our findings are supported by many previous studies. For instance, a recent study found a positive association between vitamin B_12_ intake and early vocabulary and word combination scores at 24 and 38 months, respectively, in the ALSPAC cohort.^17^ Additionally, children born to mothers with low vitamin B_12_ intake were less likely to be understood at 6 years. Preconception supplementation with vitamin B_12_ has been shown to improve cognition and language skills at 2 years of age in a randomized controlled trial,^30^ as well as to result in higher scores on expressive language at 30 months.^31^ Furthermore, elevated maternal total homocysteine levels (indicating lower vitamin B_12_ level) were associated with poorer expressive language performance in infants.^32^ However, some studies found no association between vitamin B_12_ and cognitive outcomes,^19^ or results in adverse direction—lower maternal vitamin B_12_ status was associated with higher verbal fluency scores at 9–10 years.^33^ Results concerning children’s IQ are less clear. Using the same outcome (total IQ), earlier analysis of ALSPAC data showed no association with maternal vitamin B_12_ intake.^34^ Similarly, other observational studies showed weak or no associations with intelligence or cognitive abilities.^9^^,^^35^^,^ While we did not find significant associations with total IQ either, it is noteworthy that the effect of vitamin B_12_ remained evident specifically in the verbal subtest, maintaining a consistent trend from 18 months onwards. Language development is a fundamental aspect of cognitive growth, serving both as a key of communication and a critical tool for learning and social interaction. Research has shown that early language skills significantly shape broader cognitive abilities,^36^ and are strong predictors of later academic success and social integration.^37^

### What this study adds

This study is the first large-scale study that looked at this association in Central European populations and strengthened present findings emphasizing the importance of vitamin B_12_ intake during prenatal period particularly at the time, when dietary patterns prioritizing plant sources were becoming more popular. However, given the mixed results from existing literature, our results must be interpreted with caution and respect to all limitations. Further research is needed to explore the mechanisms underlying the observed association. Additionally, future studies could investigate the optimal dose of vitamin B_12_ supplementation during pregnancy and the effects of supplementation on other health outcomes. Our categorical analysis indicates that the fourth quartile, which approached the recommended dietary reference values set by EFSA^38^ (4.5 μ/g), supports the notion that increasing vitamin B12 intake may be beneficial.

### Limitations of this study

The presented study has several limitations that should be considered when interpreting the findings. The main limitation is the use of self-reported data from FFQ, which may result in imprecise estimates of nutrient intake. As we did not have biological samples from the cohort participants, we were unable to assess the validity of the dietary measure. Additionally, FFQ captures dietary intake over a prolonged time period, which can lead to inaccuracies in assessing food intake at specific time points. Considering that brain development is influenced by the frequency and timing of nutrient intake, it is important to recognize that the impact of any nutrient deficiency can vary at different stages of development.^11^ In addition, our study employed the WISC-III to measure IQ in 8 years old children. The test was translated into Czech from the third British version of the WISC scale,^39^ lacking specific validation and standardization for the Czech children population. The observed association in our study might be influenced by the limitations of the IQ test used. Although we account for a large number of potential confounders, we cannot exclude possible effect of other factors, mainly the residual confounding of multiple nutrients and their interactions. Emphasizing the importance of adequate nutrient intake during pregnancy for neurodevelopment, it is crucial to highlight the significance of vitamin B_12_. Vitamin B_12_ plays a pivotal role in the synthesis of DNA, myelin and neurotransmitters, making it essential for proper neurological development, especially during early life.^7^ The observed associations between prenatal vitamin B_12_ intake and speech and language outcomes may be attributed to the crucial role of vitamin B_12_ in neurodevelopment. Adequate vitamin B_12_ levels during pregnancy may contribute to optimal neural connectivity and function, potentially influencing speech and language development in children.

## Conclusion

This study provided important insights into the role of maternal nutrition during pregnancy in the children’s cognitive abilities. We found consistent associations between prenatal vitamin B_12_ intake and cognitive development. The observed associations suggest that a diet low in vitamin B_12_ during pregnancy may negatively affect children’s cognitive development, particularly in speech and language. Thus, a healthy pregnancy diet with enough vitamin B_12_ sources should be emphasized.

## Supplementary Material

Supplementary_materials_revised_2_clean_fdae307

## Data Availability

The data that support the findings of this study are available from RECETOX, Faculty of Science, Masaryk University but restrictions apply to the availability of these data, which were used under licence for the current study and so are not publicly available. Data are however available from the authors upon reasonable request and with permission of RECETOX, Faculty of Science, Masaryk University.

## Notes

**Eliska Hrezova,** PhD candidate

**Gabriela Ksinan Jiskrova,** Postdoc

**Tomas Prusa,** Lecturer

**Lenka Andryskova,** Head of Population Studies Department

**Hynek Pikhart,** Professor

## References

[1] Marshall NE, Abrams B, Barbour LA. et al. The importance of nutrition in pregnancy and lactation: lifelong consequences. Am J Obstet Gynecol MFM 2021;226(5):607–32. 10.1016/j.ajog.2021.12.035 PMC9182711 34968458

[2] Cortés-Albornoz MC, García-Guáqueta DP, Velez-Van-Meerbeke A. et al. Maternal nutrition and neurodevelopment: A scoping review. Nutrients 2021;13:3530. 34684531 10.3390/nu13103530 PMC8538181

[3] Borge TC, Aase H, Brantsæter AL. et al. The importance of maternal diet quality during pregnancy on cognitive and behavioural outcomes in children: A systematic review and meta-analysis. BMJ Open 2017;7(9):e016777. 10.1136/bmjopen-2017-016777 PMC5623570 28947450

[4] Nyaradi A, Li J, Hickling S. et al. The role of nutrition in children’s neurocognitive development, from pregnancy through childhood. Front Hum Neurosci 2013;7. 10.3389/fnhum.2013.00097 PMC3607807 23532379

[5] Veena SR, Gale CR, Krishnaveni G. et al. Association between maternal nutritional status in pregnancy and offspring cognitive function during childhood and adolescence; a systematic review. BMC Pregnancy Childbirth 2016;16(1):220. 27520466 10.1186/s12884-016-1011-z PMC4982007

[6] Bath SC, Steer CD, Golding J. et al. Effect of inadequate iodine status in UK pregnant women on cognitive outcomes in their children: Results from the Avon longitudinal study of parents and children (ALSPAC). Lancet 2013;382:331–7. 23706508 10.1016/S0140-6736(13)60436-5

[7] Dror DK, Allen LH. Effect of vitamin B12 deficiency on neurodevelopment in infants: Current knowledge and possible mechanisms. Nutr Rev 2008;66(5):250–5. 18454811 10.1111/j.1753-4887.2008.00031.x

[8] Black MM . Effects of vitamin B12 and folate deficiency on brain development in children. Food Nutr Bull 2008;29(2 Suppl):S126–31. 18709887 10.1177/15648265080292S117 PMC3137939

[9] Bhate V, Deshpande S, Bhat D. et al. Vitamin B12 status of pregnant Indian women and cognitive function in their 9-year-old children. Food Nutr Bull 2008;29(4):249–54. 19227049 10.1177/156482650802900401 PMC2656635

[10] Keskin EY, Keskin M, Karaibrahimoğlu A. Association of maternal vitamin B12 status with infant findings and neurodevelopment in vitamin B12-deficient breast-fed babies. J Pediatr Hematol Oncol 2022;44(1):e91–5. 33661170 10.1097/MPH.0000000000002122

[11] Georgieff MK . Nutrition and the developing brain: Nutrient priorities and measurement. J Clin Nutr 2007;85(2):614S–20S. 10.1093/ajcn/85.2.614S 17284765

[12] Boachie J, Adaikalakoteswari A, Samavat J. et al. Low vitamin B12 and lipid metabolism: Evidence from pre-clinical and clinical studies. Nutrients 2020;12(7):1–20. 10.3390/nu12071925 PMC7400011 32610503

[13] Behere R, Deshmukh AS, Otiv S. et al. Maternal vitamin B12 status during pregnancy and its association with outcomes of pregnancy and health of the offspring: A systematic review and implications for policy in India. Front Endocrinol (Lausanne) 2021;12(288). 10.3389/fendo.2021.619176 PMC8074968 33912132

[14] Thomas S, Thomas T, Bosch RJ. et al. Effect of maternal vitamin B12 supplementation on cognitive outcomes in south Indian children: A randomized controlled clinical trial. Matern Child Health J 2019;23(2):155–63. 30003521 10.1007/s10995-018-2605-z

[15] Cruz-Rodríguez J, Díaz-López A, Canals-Sans J. et al. Maternal vitamin B12 status during pregnancy and early infant neurodevelopment: The ECLIPSES study. Nutrients 2023;15(6):1529. 36986259 10.3390/nu15061529 PMC10051123

[16] Cruz-Rodríguez J, Canals-Sans J, Hernández-Martínez C. et al. Prenatal vitamin B12 status and cognitive functioning in children at 4 years of age: The ECLIPSES study. Matern Child Nutr 2024;20(1):e13580. 10.1111/mcn.13580 PMC10750008 37938197

[17] Golding J, Gregory S, Clark R. et al. Maternal prenatal vitamin B12 intake is associated with speech development and mathematical abilities in childhood. Nutr Res 2021;86:68–78. 33551260 10.1016/j.nutres.2020.12.005 PMC7870459

[18] Villamor E, Rifas-Shiman SL, Gillman MW. et al. Maternal intake of methyl-donor nutrients and child cognition at 3 years of age. Paediatr Perinat Epidemiol 2012;26(288):328–35. 22686384 10.1111/j.1365-3016.2012.01264.x PMC3375854

[19] Boeke CE, Gillman MW, Hughes MD. et al. Choline intake during pregnancy and child cognition at age 7 years. Am J Epidemiol 2013;177(12):1338–47. 23425631 10.1093/aje/kws395 PMC3676149

[20] Wu BTF, Dyer RA, King DJ. et al. Early second trimester maternal plasma choline and betaine are related to measures of early cognitive development in term infants. PloS One 2012;7(288):7. 10.1371/journal.pone.0043448 PMC3423345 22916264

[21] Piler P, Kandrnal V, Kukla L. et al. Cohort profile: The European longitudinal study of pregnancy and childhood (ELSPAC) in the Czech Republic. Int J Epidemiol 2017;46(8):1379. 27380795 10.1093/ije/dyw091 PMC5837270

[22] Bienertová-Vašků J, Grulichová M, Mikeš O. et al. Estimated dietary iodine intake as a predictor of placental size: Evidence from the ELSPAC study. Nutr Metab (Lond) 2018;15(1):1–14. 29375646 10.1186/s12986-018-0240-8 PMC5773185

[23] NutriDatabaze.cz (2023) Databáze složení potravin České republiky. http://www.nutridatabaze.cz/ (accesed October 2023).

[24] Online potravinová databáza (2023). Slovenská internetová databáza výživového zloženia potravín. http://www.pbd-online.sk/ (October 2023, date last accessed).

[25] GOV.UK (2023). Composition of foods integrated dataset (CoFID). https://www.gov.uk/government/publications/composition-of-foods-integrated-dataset-cofid (October 2023, date last accessed).

[26] Frankenburg WK, Dodds JB. Denver developmental screening test. J Pediatr 1967;71(2):181–91. 6029467 10.1016/s0022-3476(67)80070-2

[27] Fenson L, Dale PS, Reznic S. et al. *Technical Manual for the MacArthur Communicative Development Inventories*. San Diego, CA: Developmental Psychology Laboratory, 1991.

[28] Golding J . European longitudinal study of pregnancy and childhood (ELSPAC). Paediatr Perinat Epidemiol 1989;3(4):460–9. 2587412 10.1111/j.1365-3016.1989.tb00533.x

[29] Wechsler D . *Wechsler Intelligence Scale for Children*, 3rd edn. Brno: Czech republic, Psychodiagnostika, 1996.

[30] D’souza N, Behere RV, Patni B. et al. Pre-conceptional maternal vitamin B12 supplementation improves offspring neurodevelopment at 2 years of age: PRIYA trial. Front Pediatr 2021;9:1409. 10.3389/fped.2021.755977 PMC8697851 34956975

[31] Thomas S, Thomas T, Bosch RJ. et al. Effect of maternal vitamin B12 supplementation on cognitive outcomes in south Indian children: A randomized controlled clinical trial. Matern Child Health J 2019;23(2):155–63. 30003521 10.1007/s10995-018-2605-z

[32] Srinivasan K, Thomas T, Kapanee ARM. et al. Effects of maternal vitamin B12 supplementation on early infant neurocognitive outcomes: A randomized controlled clinical trial. Matern Child Nutr 2017;13(2). 10.1111/mcn.12325 PMC6090548 27356547

[33] Veena SR, Krishnaveni G, Srinivasan K. et al. Higher maternal plasma folate but not vitamin B-12 concentrations during pregnancy are associated with better cognitive function scores in 9- to 10-year-old children in South India. J Nutr 2010;140(5):1014–22. 20335637 10.3945/jn.109.118075 PMC3672847

[34] Bonilla C, Lawlor D, Taylor A. et al. Vitamin B-12 status during pregnancy and Child’s IQ at age 8: A mendelian randomization study in the Avon longitudinal study of parents and children. PloS One 2012;7(12):e51084. 23227234 10.1371/journal.pone.0051084 PMC3515553

[35] Veena SR, Gale CR, Krishnaveni G. et al. Association between maternal nutritional status in pregnancy and offspring cognitive function during childhood and adolescence: a systematic review. BMC Pregnancy Childbirth 2016;16(1):1. 27520466 10.1186/s12884-016-1011-z PMC4982007

[36] Rose SA, Feldman JF, Jankowski JJ. A cognitive approach to the development of early language. Child Dev 2009;80(1):134–50. 19236397 10.1111/j.1467-8624.2008.01250.x PMC2780017

[37] Goldin-Meadow S, Levine SC, Lv H. et al. New evidence about language and cognitive development based on a longitudinal study: Hypotheses for intervention. Am Psychol 2014;69(6):588. 24911049 10.1037/a0036886 PMC4159405

[38] DRV Finder (2019) Dietary reference values for the EU. https://multimedia.efsa.europa.eu/drvs/index.htm. (September 2024, date last accessed)

[39] Wechsler D, Golombok S, Rust J. *WISC-CN2IUK Wechsler Intelligence Scale for Children—Third Edition UK Manual*. UK: Psychological Corporation, Sidcup, 1992.

