# Supplementary material for: Vitamin B12 intake during pregnancy linked to child speech development and intelligence quotient
Source: J Public Health (Oxf). 2024 Dec 14;47(1):34–44. doi: 10.1093/pubmed/fdae307 (PMC11879010; doi:10.1093/pubmed/fdae307)
Supplement: Supplementary_materials_revised_2_clean_fdae307 [file supplementary_materials_revised_2_clean_fdae307.docx]

# Supplementary materials

Table S1 Selected food groups for estimation of vitamin B12 intake

| List of food group | Food item |
| --- | --- |
| Breakfast cereals | Oatmeal, puffed rice, popcorn, porridge, flavoured granola, bran, grain sprouts |
| Kolaches, sweet rolls | Sweet rolls, kolaches, cakes, soufflés, leavened cakes, sponge cake, doughnuts, desserts, omelettes |
| Biscuits, wafers, sponge biscuits | Sponge biscuits, filled wafers |
| Puddings | Fruit-based, milk-based, fruit yoghurt, whipped cottage cheese, products |
| Poultry | Chicken, ducks, goose |
| Red meat | Beef, pork, mutton, ham, bacon |
| Pâtés | Pâtés, minced meat |
| Offal | Liver, kidney, heart |
| Sausages | Smoked meat, frankfurters, sausages, other smoked meat products, hamburgers, hamburger patties |
| Pizza | |
| Fish | Canned fish, fresh fish, frozen fish |
| Shellfish | Oysters, crabs, snails |
| Eggs | |
| Cheese | |
| Boiled potatoes | Boiled potatoes, mashed potatoes |
| Chocolate | Chocolate, confectionery |
| Milk | Milk, dairy products |

Table S2 Model 2 (fully adjusted) for speech and language outcomes at 18 months with all the covariates

| Variable | | Language | | Talking and understanding | |
| --- | --- | --- | --- | --- | --- |
|  |  | B [95% CI] | P value | B [95% CI] | P value |
| Sex of the child | Male | -1.71 [-2.05; -1.39] | <0.001 | -15.37 [-18.95; -11.80] | <0.001 |
| Birth weight | < 2,500 | -0.44 [-1.19; 0.32] | 0.258 | -6.20 [-14.17; 1.76] | 0.127 |
|  | > 2,500 | 1 |  | 1 |  |
| Head circumference | | 0.32 [0.20; 0.45] | <0.001 | 3.92 [2.57; 5.27] | <0.001 |
| Maternal age | | -0.08 [-0.11; -0.04] | <0.001 | -0.56 [-0.99; -0.13] | 0.010 |
| BMI prior to pregnancy | | -0.02 [-0.08; 0.04] | 0.490 | -0.02 [-0.62; 0.58] | 0.959 |
| Education – mother | Primary | 0.44 [-0.57; 1.45] | 0.393 | 12.75 [1.72; 23.78] | 0.024 |
|  | Vocational | -0.15 [-0.84; 0.54] | 0.668 | 2.91 [-4.18; 10.00] | 0.419 |
|  | Secondary | 0.29 [-0.27; 0.84] | 0.312 | 4.54 [-1.33; 10.41] | 0.129 |
|  | University | 1 |  | 1 |  |
|  | P trend |  | 0.899 |  | 0.120 |
| Education – father | Primary | -0.37 [-1.42; 0.67] | 0.483 | -13.37 [-24.70; -2.05] | 0.021 |
|  | Vocational | -0.49 [-1.01; 0.04] | 0.069 | -6.67 [-11.16; -1.17] | 0.018 |
|  | Secondary | -0.15 [-0.68; 0.38] | 0.571 | -5.17 [-10.69; 0.35] | 0.066 |
|  | University | 1 |  | 1 |  |
|  | P trend |  | 0.084 |  | 0.004 |
| Alcohol consumption during the first 3 months of pregnancy | Yes | 0.01 [-0.39; 0.40] | 0.979 | -1.60 [-5.90; 2.70] | 0.464 |
|  | No | 1 |  |  |  |
| Smoking status during the pregnancy | Smoker | 0.02 [-0.71; 0.76] | 0.949 | -2.60 [-11.16; 5.96] | 0.549 |
|  | Ex-smoker | 0.46 [0.02; 0.90] | 0.039 | 3.90 [-0.80; 8.60] | 0.103 |
|  | Non-smoker | 1 |  | 1 |  |
| Dietary supplements during the pregnancy | No | -0.21 [-0.58; 0.15] | 0.245 | -2.42 [-6.34; 1.51] | 0.226 |
|  | Yes | 1 |  | 1 |  |
| Total energy intake | | -0.08 [-0.20; 0.04] | 0.245 | -0.93 [-2.21; 0.35] | 0.154 |
| Adults (including mother) in the same household | | 0.25 [0.06; 0.44] | 0.009 | 2.94 [1.03; 4.86] | 0.003 |
| Other children in the same household | | -0.32 [-0.55; -0.09] | 0.007 | -1.86 [-4.47; 0.74] | 0.160 |
| Working status – mother* | Yes | -0.60 [-1.27; 0.07] | 0.079 | -4.17 [-11.45; 3.10] | 0.260 |
|  | No | 1 |  | 1 |  |
| Breast feeding status* | No | -0.47 [-1.08; 0.15] | 0.140 | -5.85 [-12.29; 0.59] | 0.075 |
|  | Yes | 1 |  | 1 |  |

_* reported at the child’s 18-month follow-up_

Table S3 Model 2 (fully adjusted) for IQ verbal at 8 years with all the covariates

| Variable | | B [95% CI] | P value |
| --- | --- | --- | --- |
| Sex of the child | Male | -0.33 [-2.57; 1.91] | 0.775 |
| Birthweight | < 2,500 | -2.51 [-7.95; 2.93] | 0.365 |
|  | > 2,500 | 1 |  |
| Head circumference | | 1.12 [0.19; 2.05] | 0.018 |
| Maternal age | | 0.04 [-0.24; 0.32] | 0.772 |
| BMI prior to pregnancy | | -0.09 [-0.50; 0.32] | 0.656 |
| Education – mother | Primary | -3.82 [-11.39; 3.75] | 0.322 |
|  | Vocational | -8.48 [-12.77; -4.18] | <0.001 |
|  | Secondary | -3.21 [-6.59; 0.17] | 0.063 |
|  | University | 1 |  |
|  | P trend |  | 0.033 |
| Education – father | Primary | -7.55 [-16.70; 1.61] | 0.105 |
|  | Vocational | -7.28 [-10.77; -3.78] | <0.001 |
|  | Secondary | -4.27 [-7.86; -0.68] | 0.020 |
|  | University | 1 |  |
|  | P trend |  | <0.001 |
| Alcohol consumption during the first 3 months of pregnancy | Yes | -1.14 [-3.79; 1.52] | 0.402 |
|  | No | 1 |  |
| Smoking status during the pregnancy | Smoker | 1.09 [-4.42; 6.60] | 0.698 |
|  | Ex-smoker | 0.03 [-2.91; 2.96] | 0.986 |
|  | Non-smoker | 1 |  |
| Dietary supplements during the pregnancy | No | 1.29 [-1.28; 3.86] | 0.323 |
|  | Yes | 1 |  |
| Total energy intake | | -0.06 [-0.86; 0.74] | 0.878 |
| Adults (including mother) in the same household | | -0.62 [-1.95; 0.70] | 0.356 |
| Other children in the same household | | -1.75 [-3.59; 0.08] | 0.061 |
| Breast feeding status* | No | -1.05 [-5.55; 3.45] | 0.649 |
|  | Yes | 1 |  |
| Working status – mother* | Yes | -1.19 [-5.64; 3.27] | 0.602 |
|  | No | 1 |  |

_* reported at the child’s 18-month follow-up_

Table S4 The effect of vitamin B12 intake in quartiles on speech and language tests outcomes in children

|  | Model 0 | | | | | Model 2 | | | | |
| --- | --- | --- | --- | --- | --- | --- | --- | --- | --- | --- |
| Test | B12 intake (µg/day) | | | | | B12 intake (µg/day) | | | | |
|  | Q1  (<1.84) | Q2  (1.85-2.76) | Q3  (2.77-4.01) | Q4 (>4.01) | Trend  p value | Q1  (<1.84) | Q2  (1.85-2.76) | Q3  (2.77-4.01) | Q4 (>4.01) | Trend  p value |
|  | B [95% CI] | B [95% CI] | B [95% CI] |  |  | B [95% CI] | B [95% CI] | B [95% CI] |  |  |
| 18-month language | **-0.52 [-0.99; -0.04]** | -0.15 [-0.62; 0.33] | -0.29 [-0.74; 0.17] | 1 | 0.068 | **-0,76 [-1,35; -0,17]** | -0.31 [-0.85; 0.23] | -0.43 [-0.91; 0.04] | 1 | 0.031 |
| 18-month talking and understanding | **-5.70 [-10.88; -0.51]** | -3.89 [-8.82; 1.03] | -4.46 [-9.45; 0.53] | 1 | 0.044 | **-8.30 [-14.29; -2.31]** | -5.61 [10.91; -0.30] | **-5.79 [-10.97; -0.61]** | 1 | 0.011 |

_Model 0 unadjusted. Model 2 adjusted for mother´s age, mother´s BMI prior to pregnancy, sex of the child, birthweight, head circumference, alcohol consumption during the first 3 months of pregnancy, smoking status during the pregnancy, total energy intake and dietary supplement use during pregnancy, maternal and paternal education, number of children younger than 15 years of age in the same household, number of adults over 18 years of age in the same household, breast feeding reported at 18th month of children´s age, and if the mother worked at 18th month of children´s age._

Table S5 The effect of vitamin B12 intake in quartiles on intelligibility tests outcomes in children

|  | Model 0 | | | | | Model 2 | | | | |
| --- | --- | --- | --- | --- | --- | --- | --- | --- | --- | --- |
| Test | B12 intake (µg/day) | | | | | B12 intake (µg/day) | | | | |
|  | Q1  (<1.84) | Q2  (1.85-2.76) | Q3  (2.77-4.01) | Q4  (>4.01) | Trend  p value | Q1  (<1.84) | Q2  (1.85-2.76) | Q3  (2.77-4.01) | Q4  (>4.01) | Trend  p value |
|  | B [95% CI] | B [95% CI] | B [95% CI] |  |  | B [95% CI] | B [95% CI] | B [95% CI] |  |  |
| 3-year intelligibility | **0.80 [0.67; 0.95]** | 0.93 [0.79; 1.11] | 1.01 [0.86; 1.20] | 1 | 0.008 | 0.87 [0.72; 1.04] | 0.96 [0.81; 1.15] | 1.05 [0.88; 1.24] | 1 | 0.083 |
| 5-year intelligibility | 0.92 [0.77; 1.09] | 1.03 [0.86; 1.23] | 1.06 [0.89; 1.26] | 1 | 0.301 | 0.85 [0.67; 1.09] | 0.96 [0.76; 1.23] | 0.93 [0.73; 1.19] | 1 | 0.649 |
| 7-year intelligibility | 0.93 [0.78; 1.11] | 1.06 [0.89; 1.27] | 1.17 [0.99; 1.39] | 1 | 0.262 | 0.98 [0.82; 1.18] | 1.07 [0.90; 1.29] | 1.21 [1.02; 1.43] | 1 | 0.595 |

_Model 0 unadjusted. Model 2 adjusted for mother´s age, mother´s BMI prior to pregnancy, sex of the child, birthweight, head circumference, alcohol consumption during the first 3 months of pregnancy, smoking status during the pregnancy, total energy intake and dietary supplement use during pregnancy, maternal and paternal education, number of children younger than 15 years of age in the same household, number of adults over 18 years of age in the same household, breast feeding reported at 18th month of children´s age, and if the mother worked at 18th month of children´s age._

Tables S6 The effect of vitamin B12 intake in quartiles on intelligence tests outcomes in children

|  | Model 0 | | | | | Model 2 | | | | |
| --- | --- | --- | --- | --- | --- | --- | --- | --- | --- | --- |
| Test | B12 intake (µg/day) | | | | | B12 intake (µg/day) | | | | |
|  | Q1  (<1.89) | Q2  (1.89-2.81) | Q3  (2.82-4.09) | Q4 (>4.09) | Trend  p value | Q1  (<1.89) | Q2  (1.89-2.81) | Q3  (2.82-4.09) | Q4 (>4.09) | Trend  p value |
|  | B [95% CI] | B [95% CI] | B [95% CI] |  |  | B [95% CI] | B [95% CI] | B [95% CI] |  |  |
| 8-year IQ verbal | **-7.08 [-10.53; -3.63]** | **-4.13 [-7.56; -0.69]** | **-3.47 [-6.93; -0.01]** | 1 | <0.001 | **-5.74 [-9.74; -1.75]** | -3.61 [-7.30; 0.07] | -2.42 [-5.88; 1.03] | 1 | 0.005 |
| 8-year IQ perform | **-5.40 [-8.93; -1.87]** | -2.23 [-5.78; 1.32] | -0.95 [-4.52; 2.61] | 1 | 0.002 | -2.63[-6.66; 1.40] | -0.38 [-4.20; 3.45] | 0.88 [-2.77; 4.52] | 1 | 0.115 |
| 8-year IQ total | **-6.66 [-9.98; -3.34]** | **-3.50 [-6.84; -0.16]** | -2.53 [-5.87; 0.81] | 1 | <0.001 | **-4.94 [-8.71; -1.16]** | -2.60 [-6.15; 0.94] | -1.13 [-4.46; 2.21] | 1 | 0.008 |

_Model 0 unadjusted. Model 2 adjusted for mother´s age, mother´s BMI prior to pregnancy, sex of the child, birthweight, head circumference, alcohol consumption during the first 3 months of pregnancy, smoking status during the pregnancy, total energy intake and dietary supplement use during pregnancy, maternal and paternal education, number of children younger than 15 years of age in the same household, number of adults over 18 years of age in the same household, breast feeding reported at 18th month of children´s age, and if the mother worked at 18th month of children´s age._
